# Associations Between Plasma Levels of NLRP3 Protein, Interleukin-1 Beta and Features of Acute ST-Elevation Myocardial Infarction

**DOI:** 10.3390/jpm14111103

**Published:** 2024-11-13

**Authors:** Vyacheslav Ryabov, Yulia Samoilova, Aleksandra Gombozhapova, Anastasiia Nesova, Irina Kologrivova

**Affiliations:** 1 Department of Cardiac Emergency, Cardiology Research Institute, Tomsk National Research Medical Center, Russian Academy of Sciences, 634012 Tomsk, Russia; rvvt@cardio-tomsk.ru (V.R.); samoylova.ssmu@yandex.ru (Y.S.); gombozhapova.ae@ssmu.ru (A.G.); 2 Department of Clinical Laboratory Diagnostics, Cardiology Research Institute, Tomsk National Research Medical Center, Russian Academy of Sciences, 634012 Tomsk, Russia; kiv@cardio-tomsk.ru

**Keywords:** acute myocardial infarction, postinfarction inflammation, NLRP3 inflammasome, interleukin-1β

## Abstract

Background. Phenotyping inflammation in ST-elevation myocardial infarction (STEMI) is a challenge for modern cardiology. NLRP3 inflammasome is a proven predictor of adverse outcomes in cardiovascular disease, but its specificity in stratifying inflammatory activity in patients with myocardial infarction (MI) has not been demonstrated. The aim of this paper is to describe the levels of NLRP3 protein and IL-1β concentrations and their changes in dynamics and associations with clinical, laboratory and instrumental characteristics of patients with STEMI. Methods. A total of 45 patients with STEMI were enrolled. Concentrations of NLRP3 and IL-1β were evaluated in arterial and venous EDTA blood from the infarct-related coronary and peripheral arteries and veins on days 1, 3 and 7 after MI. Results and Conclusions. The concentrations of markers were higher on the first day after MI with a maximum decrease on the third day. The levels of both markers in venous plasma correlated with those in arterial blood, allowing their routine determination in venous plasma on the first day after MI. IL-1β levels correlated directly with the wall motion index and inversely with left ventricular ejection fraction and stroke volume, which characterize the potential contribution to adverse myocardial remodeling. There were two multidirectional trends in changes in NLRP3 and IL-1β levels during hospitalization. Initially higher levels with a gradual decrease by day 7 were associated with a longer duration of myocardial ischemia and higher plasma troponin I levels. Further evaluation of the long-term outcomes of MI will allow identifying inflammatory factors that input to the development of secondary major adverse cardiac events and will provide a new step in the understanding of inflammatory phenotyping.

## 1. Introduction

Acute myocardial infarction (MI) is the most severe manifestation of coronary heart disease (CHD), accounting for more than one-third of all deaths in developed countries [1]. Risk factor modification, the widespread use of evidence-based medical therapies, and active introduction of timely myocardial reperfusion techniques into practice have led to a significant reduction in hospital mortality in patients with MI [2]. However, a significant proportion of patients experience major adverse cardiac events (MACE) in the time period following the index hospitalization. The Swedish national registry (*n* = 108,315) demonstrated that 18.3% of patients had MACE in the first year after MI, with one in five patients experiencing adverse cardiac events in the following three years [3]. In the UK–Belgium GRACE study (*n* = 3721), the incidence of MACE within 5 years of discharge was 31.2% in patients with non-ST elevation MI, 27.2% in patients with ST elevation MI (STEMI) and 23.7% in patients with unstable angina [4]. Thus, the search for predictors of adverse remote outcomes and for mechanisms influencing them remains relevant in the context of the overall burden of cardiovascular diseases (CVD) reduction.

In recent years, the inflammatory theory of the development, progression and adverse outcome of CVD has become a subject of increasing scientific interest. According to this theory, progressive lipid deposition in the arterial endothelium and subsequent thrombosis are associated with the activity of inflammatory processes in the atherosclerotic plaque and surrounding tissues [5].

The development of secondary MACE is now explained by the concept of ‘residual inflammatory risk’. Phenotyping inflammation in acute MI represents a current challenge [6]. There is substantial evidence that NLRP3 inflammasome-associated inflammation is important in the initiation and progression of atherosclerosis [7]. It is also suggested that the NLRP3 inflammasome, as one of the key links in the signaling cascade of the inflammatory process, may determine the degree of residual inflammatory risk [5], but its specificity in stratifying inflammatory activity in patients with MI has not been demonstrated. The NLRP3 inflammasome consists of the Nod-like receptor family pyrin domain containing 3 (NLRP3), caspase-1 and an apoptosis-associated speck-like protein containing the caspase recruitment domain (ASC protein). NLRP3 inflammasome activation occurs under the influence of modified lipoproteins, cholesterol crystals, lipopolysaccharides and reactive oxygen species. The synthesis of interleukin-1 beta (IL-1β), interleukin-6 (IL-6) and interleukin-18 (IL-18) is triggered by the NLRP3 inflammasome and is associated with the activation of caspase-1 [5]. IL-1β is a known pro-inflammatory cytokine that has been shown in several studies to adversely affect the course of the postinfarction period [8].

To date, the obtained experimental data have not been translated into clinical practice. There are no studies investigating the associations between the levels of these biomarkers and clinical-instrumental and laboratory characteristics. Finally, there are no data to answer the question of whether patients can be stratified on the basis of different levels of these biomarkers to predict the degree of residual inflammatory risk.

The aim of the present study is to translate the data from experimental studies into clinical practice and to describe the levels of NLRP3 protein and IL-1β concentrations, their changes in dynamics and their associations with clinical, laboratory and instrumental characteristics of patients with STEMI.

## 2. Materials and Methods

The study included 45 patients admitted to the emergency cardiology department in 2022–2023. The inclusion criteria were STEMI type 1 and the development of symptoms of acute myocardial ischemia within 24 h prior to hospital admission. The study was approved by the Ethical Committee of the Cardiology Research Institute of the Tomsk National Research Medical Centre, protocol No. 225 of 9 February 2022. All patients completed an informed consent form to participate in the study.

The study material was arterial and venous blood plasma samples from three different vascular basins. Blood samples were taken from the infarct-related coronary artery (IRA) and the peripheral (radial/femoral) artery in EDTA tubes during percutaneous coronary intervention (PCI) on the first day of MI for the evaluation of NLRP3 and IL-1β concentrations. Venous blood samples were collected in EDTA tubes during the first days of MI before PCI and on the third and seventh days of MI. The blood samples were centrifuged (2700 rpm) for 10 min, the obtained plasma was aliquoted and the samples were frozen at −80 °C for further study. The concentration of NLRP3 and IL-1β in blood plasma was evaluated by enzyme-linked immunosorbent assay (ELISA) (FineTest, Wuhan, China). Results were counted photometrically using an INFINITE F50 microplate analyzer (Tecan, Männedorf, Switzerland) at a wavelength of 450 nm with a reference wavelength of 620 nm. Results were expressed in ng/mL for NLRP3 and in pg/mL for IL-1β. The sensitivity of the method was 0.469 ng/mL for NLRP3 and 2.344 pg/mL for IL-1β.

The statistical processing of the results was performed using the licensed version of Statistica V12.0 for Windows. Quantitative parameters were described as median (Me) and interquartile range (Q25; Q75) or mean and standard deviation (M ± SD); qualitative parameters were described as absolute values (*n*) and relative frequencies (%). The Shapiro–Wilk criterion was used to assess the normality of the distribution of the variables in the sample. The Kruskal–Wallis test was used to compare quantitative indicators between groups of independent samples. The non-parametric Mann–Whitney test was used to assess differences between independent samples and the Wilcoxon test between dependent samples. The Spearman correlation coefficient was used to reveal correlations between variables. Values *p* < 0.05 were considered statistically significant.

## 3. Results

### 3.1. General Characteristics of the Patients

The majority of patients were men (69%). The mean age was 58 (49; 68) years. The mean total duration of myocardial ischemia was 213 (141; 492) minutes. The main clinical and anamnestic characteristics of the patients with MI enrolled in the study are shown in Table 1 and Table 2.

### 3.2. NLRP3 Protein and IL-1β Levels in Different Vascular Basins and Their Changes During Hospitalization

**Figure 1:**
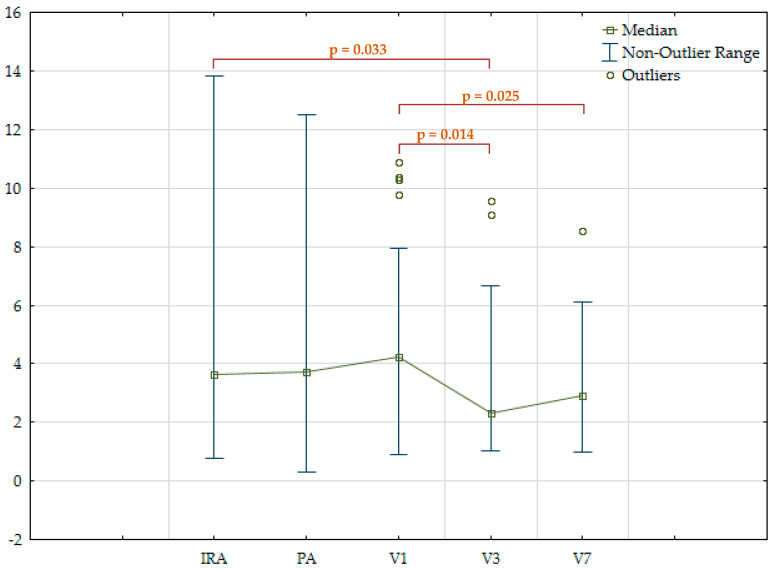
NLRP3 protein plasma levels (ng/mL) in all patients. NLRP3—Nod-like receptor family pyrin domain containing 3; IRA—infarct-related coronary artery; PA—peripheral artery; V1—peripheral vein, day 1; V3—peripheral vein, day 3; V7—peripheral vein, day 7.

**Figure 2:**
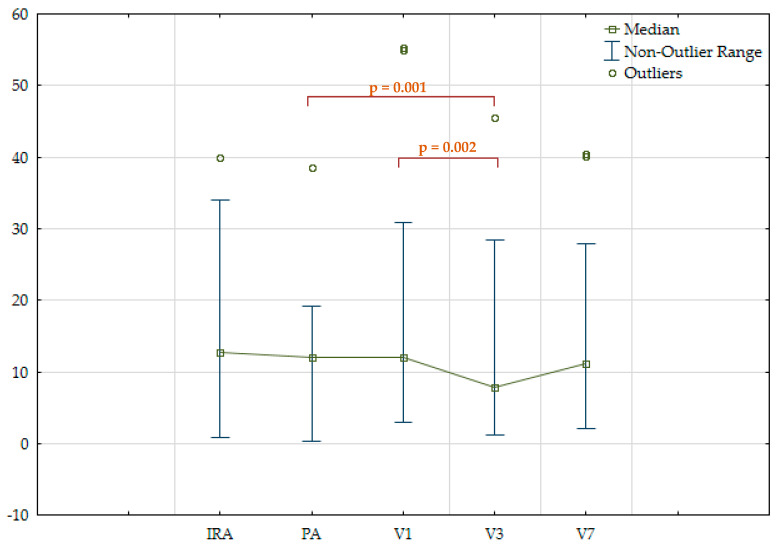
IL−1 beta plasma levels (pg/mL) in all patients. IRA—infarct-related coronary artery; PA—peripheral artery; V1—peripheral vein, day 1; V3—peripheral vein, day 3; V7—peripheral vein, day 7.

Concentrations of NLRP3 protein and IL-1β were measured in all three vascular basins on different days of hospitalization. A moderate direct correlation was found between IL-1β and NLRP3 protein levels in IRA plasma (r = 0.305; *p* = 0.049) and peripheral artery plasma (r = 0.403; *p* = 0.005). When assessing the NLRP3 protein and IL-1β in the total sample of patients, a tendency for their concentrations to decrease with the duration of hospitalization was observed. The most pronounced decrease in both markers was observed in venous blood plasma on the third day after MI. A statistically significant difference was found between NLRP3 protein and IL-1β levels in venous blood plasma on day 3 and in IRA and peripheral artery plasma, respectively. In addition, there was a slight increase in the levels of the markers on day seven compared to day three (Table 3; Figure 1 and Figure 2).

**Figure 3:**
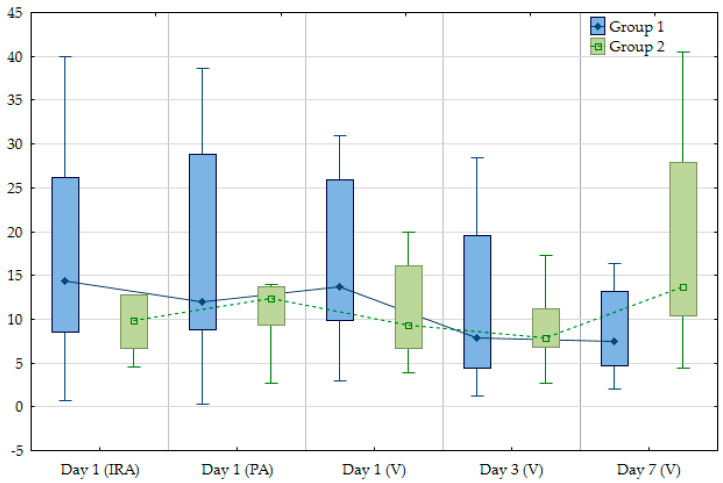
Main trends in IL−1 beta concentrations (pg/mL) in arterial and venous blood plasma from different vascular basins during hospitalization. IRA—infarct-related coronary artery; PA—peripheral artery; V—peripheral vein.

**Figure 4:**
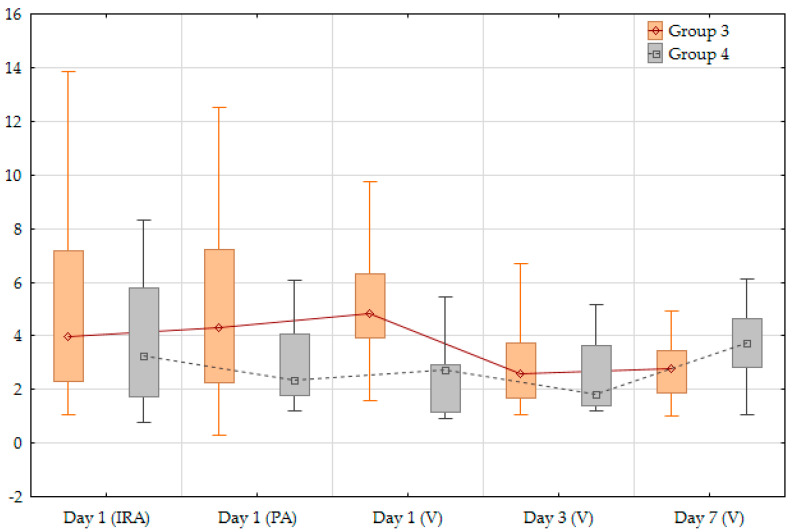
Main trends in NLRP3 protein concentrations (ng/mL) in plasma arterial and venous blood from different vascular basins during the hospital period. IRA—infarct-related coronary artery; PA—peripheral artery; V—peripheral vein.

It is important to note that not all patients had the same dynamics of change in biomarker concentrations. Any changes in the concentrations of both markers were taken as the dynamics of their levels with correction for the sensitivity of the method used. Two main trends in biomarker levels were identified among the patients: (1) initially higher levels of NLRP3 protein or IL-1β with a gradual decrease by the seventh day with a minimum level on the third day (groups 1 and 3); (2) initially lower levels of biomarkers with a minimum concentration on the third day followed by a more significant increase by the seventh day (groups 2 and 4) (Figure 3 and Figure 4).

The first mode of concentration changes was typical for the majority of patients, both for IL-1β (21; 58.3%) and NLRP3 protein (30; 71.4%). The dynamics of change in both markers in venous blood on day 3 were statistically significant (*p* < 0.05). The groups were comparable with the main clinical, laboratory and instrumental characteristics of myocardial ischemic damage (Table 4 and Table 5).

Statistically significant differences were found with regard to the dynamics of IL-1β levels: in group 1, the total time of myocardial ischemia was longer, and higher levels of high-sensitivity troponin-I were registered on days 3 and 7. Group 1 also showed a trend towards a lower left ventricular ejection fraction (LVEF) on day 1, with a concomitant increase in the wall motion index (WMI) and end-systolic volume (ESV). To assess WMI, the left ventricle was conditionally divided into 17 segments, each of which were assigned a numerical value from 1 to 4, depending on the presence and degree of local contractility impairment: 1—normal; 2—hypokinesia; 3—akinesia; 4—dyskinesia. The scores were summed and divided by the number of myocardial segments visualized. Overall, the observed differences suggest more pronounced myocardial damage in group 1 compared to group 2. No statistically significant differences were found when comparing the characteristics of groups 3 and 4.

### 3.3. Comparison of NLRP3 Protein and IL-1β Concentrations in Plasma from Different Vascular Basins

**Figure 5:**
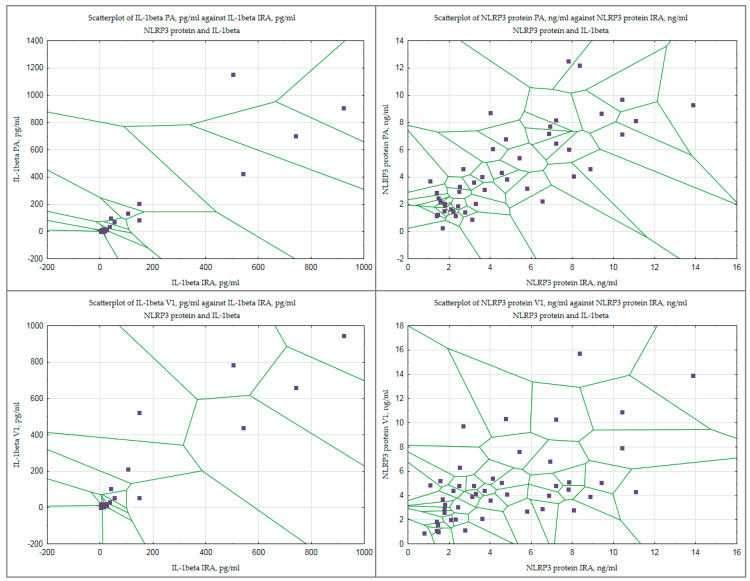
Correlations of NLRP3 and IL−1 beta protein levels in arterial and venous blood plasma at different time points. Note: NLRP3—Nod-like receptor family pyrin domain containing 3; IRA—infarct-related coronary artery; PA—peripheral artery; V1—peripheral vein, day 1. The data presented using Voronoi scatterplot.

The arterial and venous blood plasma levels of the studied pro-inflammatory markers were compared (Table 6 and Figure 5).

With regard to IL-1β, strong correlations were found between its levels in the plasma of IRA and peripheral arterial blood (r = 0.774; *p* = 0.001), as well as between IRA and venous blood on day 1 (r = 0.658; *p* = 0.037) and day 3 (r = 0.722; *p* = 0.005). There was a strong correlation between IL-1β levels in peripheral arterial and venous plasma on day 3 (r= 0.733; *p* = 0.019) and day 7 (r= 0.608; *p* = 0.001). In relation to NLRP3 protein levels, strong correlations were found between the levels of this marker in IRA and peripheral arterial plasma (r = 0.793; *p* = 0.003) and between peripheral arterial and venous plasma levels on day 1 (r = 0.682; *p* = 0.001).

### 3.4. The Associations of NLRP3 Protein and IL-1β Levels with Clinical and Laboratory Characteristics of the Patients

According to the results of the study, there were no differences in the levels of these pro-inflammatory markers in patients with primary and recurrent MI, nor were there any differences according to age, sex, smoking, dyslipidemia, the location of the MI and the presence of type 2 diabetes mellitus.

Unexpectedly, in obese patients, IL-1β levels were significantly lower in plasma obtained from the IRA, peripheral artery and vein on the first day of MI. On further analysis, we detected an inverse correlation between obesity of any degree and IL-1β concentration on the first day in blood plasma from all three vessels (IRA, peripheral artery and vein).

Moderate inverse correlations were observed between body mass index (BMI) and IL-1β levels in IRA (r = −0.545; *p* = 0.0002), peripheral arterial (r = −0.461; *p* = 0.002) and venous blood plasma obtained on the first (r = −0.464; *p* = 0.002), third (r = −0.332; *p* = 0.045) and seventh (r = −0.340; *p* = 0.042) day after MI.

There was a strong direct correlation between IL-1β levels in venous blood plasma on day 1 of MI and C-reactive protein (CRP) levels (r = 0.681; *p* = 0.010) and a less pronounced correlation with peripheral blood leukocyte levels (r = 0.336; *p* = 0.004). As with IL-1β levels, there was a direct correlation between NLRP3 protein levels in venous blood plasma on day 1 of MI and peripheral blood leukocyte levels (r = 0.306; *p* = 0.004), but no correlation with CRP levels.

The level of NLRP3 protein in patients with chronic kidney disease (CKD) was higher than in patients without CKD at all time points. There was a moderate direct correlation between the presence of CKD and the level of NLRP3 protein in IRA (r = 0.348; *p* = 0.019), peripheral arterial (r = 0.378; *p* = 0.011) and venous blood plasma obtained on day 1 (r = 0.439; *p* = 0.006), day 3 (r = 0.330; *p* = 0.030) and day 7 (r = 0.554; *p* = 0.0002) of MI. There was also a direct correlation between NLRP3 protein levels and venous blood plasma urea levels obtained on day 1 (r = 0.451; *p* = 0.010) and day 7 (r = 0.600; *p* = 0.0004) of MI.

### 3.5. Associations of NLRP3 Protein, IL-1β Levels with Characteristics of Myocardial Injury in All Patients

A separate area of interest in this study was the investigation of the relationships between NLRP3 protein and IL-1β concentrations in different vascular basins and laboratory and instrumental parameters characterizing acute MI. To assess the degree of myocardial ischemic damage, the following data were considered: clinical (total time of myocardial ischemia; acute heart failure; life-threatening arrhythmias), laboratory (high-sensitivity troponin I level) and instrumental (blood flow at TIMI baseline and at the time of PCI; end-diastolic volume; ESV; LVEF; stroke volume (SV); cardiac index; WMI). In the course of further analysis, no strong correlations (both direct and inverse) were found between the above parameters and NLRP3 protein and IL-1β levels in the total sample of patients. There were moderate direct correlations between WMI values and IL-1β levels in IRA (r = 0.441; *p* = 0.0001), peripheral artery (r = 0.356; *p* = 0.0018) and venous blood on day 1 (r = 0.326; *p* = 0.0002). Moderate inverse correlations were found for IL-1β concentration in the IRA and LVEF (r = −0.336; *p* = 0.0004) and SV (r = −0.369; *p* = 0.0016).

When the correlations between NLRP3 protein levels and the above mentioned MI characteristics were examined, a moderate direct relationship was found between NLRP3 protein levels in venous blood plasma on day 7 and baseline blood flow in the IRA (r = 0.441; *p* = 0.004).

### 3.6. Patients with MI Complicated by Cardiogenic Shock

From the total number of patients in the main group, patients with MI complicated by cardiogenic shock (CS) were selected (6; 13.3%). There were strong direct correlations between WMI and plasma IL-1β levels in the IRA (r = 0.894; *p* = 0.0014), peripheral artery (r = 0.704; *p* = 0.008) and vein on day 1 (r = 0.763; *p* = 0.024) in patients with CS. This conclusion requires further confirmation considering the small number of CS patients in our study. The exclusion of patients with CS from the analysis did not affect the results described above, which were obtained for the total sample of patients with MI.

## 4. Discussion

It is well known that inflammation potentiates the adverse course and progression of CVD while at the same time being a prerequisite condition for repair processes [9,10]. However, it becomes clear that the current knowledge of the cellular and molecular mechanisms of inflammation in atherosclerosis is inadequate, and subphenotypes of inflammation during STEMI are highly heterogeneous and have not yet been studied. In this context, clinical research aimed at identifying the most specific targets of the inflammatory cascade in different cardiovascular pathologies and clinical stratification of patients according to the levels of markers and their dynamics in the blood remain relevant.

The results of recent clinical trials indicate the great potential of anti-inflammatory strategies in preventing the progression of atherosclerosis and its complications [11,12,13]. The anticipated negative role of the NLRP3 inflammasome in adverse myocardial remodeling has led to the identification of this marker as a potential therapeutic target to reduce the risk of CHD and its complications. Basic research has also demonstrated the negative role of the NLRP3 inflammasome in the pathogenesis and progression of CHD, making the study of NLRP3 inflammasome-associated inflammation in MI highly relevant [5]. The NLRP3-mediated pathway is one of the mechanisms of IL-1β activation [14]. The negative role of IL-1β has been demonstrated at later time points after MI, but its role in the acute phase is less clear [6]. It is worth noting that most of the experimental data available to date has been generated in mouse models [14] and has not been translated into clinical trials.

To our knowledge, this is the first study to investigate the quantitative levels of circulating pro-inflammatory markers such as NLRP3 protein and IL-1β in patients with STEMI in various vascular basins. There are currently very few randomized clinical trials assessing levels of the NLRP3 inflammasome and pro-inflammatory cytokines in the plasma of patients with STEMI. For example, a recent study evaluated levels of the NLRP3 inflammasome and IL-1β in patients with MI and depression [15]. The IL-1β levels obtained are similar to those in our study. However, the levels of the NLRP3 inflammasome were slightly higher in the patients in our study, which may be due to the fact that our sample is more non-selective. The psychological status of the patients also was not evaluated in our study, which could have influenced its results.

In previous studies, the levels of NLRP3 protein and IL-1β have only been studied in the venous system, but not in arterial blood [15,16]. The levels of these markers measured directly in the IRA may more accurately reflect their influence on the characteristics of acute ischemic myocardial injury. We are the first to demonstrate that the valuation of plasma levels of NLRP3 protein and IL-1β is possible in circulating blood of different vascular basins. The levels of both markers in venous plasma were similar to those in peripheral arterial blood and IRA. This finding has important implications for the possibility of NLRP3 protein and IL-1β concentrations measurement in venous plasma in routine cardiology practice. Taking into account the minimal concentrations of both markers on day 3, blood sampling to determine the levels of inflammatory markers should be performed early in the course of MI (day 1).

In the present study, we were able to assess the levels and patterns of changes in plasma concentrations of NLRP3 protein and IL-1β and identify some associations with the degree of myocardial injury. For example, direct correlations were observed between WMI values and IL-1β levels on day 1 in both the arterial and venous beds. The WMI has previously been shown to comprehensively reflect the extent of left ventricular myocardial damage and is used in practice for the semi-quantitative assessment of local contractility impairment [17]. It should be noted that we did not assess the extent of myocardial damage using cardiac magnetic resonance imaging (MRI), which is a weakness of our study. The IL-1β levels were also inversely correlated with such parameters as LVEF and SV. The data obtained may characterize the contribution of IL-1β to adverse myocardial remodeling after myocardial infarction. No significant correlations were found between NLRP3 protein levels and characteristics of myocardial injury.

An important aspect of the study was the demonstration of dynamic changes in the concentrations of both markers during hospital follow-up and the heterogeneity of these dynamics for different groups of patients. Thus, we described two main trends in dynamic changes on the basis of which the patients were divided into groups. It is possible that with a larger number of patients included in the analysis in the future, the phenotypic pattern of marker dynamics would have been represented by additional variants. In general, it was found that the group with a decrease in IL-1β concentration by day 7 was characterized by more severe myocardial damage, which can be assessed on the basis of clinical and laboratory data. This finding is consistent with the direct correlations between IL-1β levels and WMI, as well as the inverse correlations with LVEF and SV in the total patients’ sample.

The pleiotropic effects of inflammatory markers are known to be a serious obstacle to the development of effective standard anti-inflammatory treatments [18]. There is evidence that IL-1β may play an important role in repair processes after MI [19]. The involvement of IL-1β has also been shown to be necessary for the activation of the reparative phenotype of macrophages, which mediate tissue regeneration processes [20]. In addition, there are studies showing that the absence of NLRP3 results in increased myocardial infarct size after in vivo ischemia–reperfusion [21,22].

Given the unequal dynamics of plasma levels of IL-1β and NLRP3 protein observed in our study and their known pleiotropic effects, it is reasonable to assume that the potential use of anti-inflammatory treatment will have a different effect in subacute and remote periods of MI in different patients. In addition, some patients may be harmed by anti-inflammatory treatment. At this stage of the work, we did not compare trends in changes in the concentrations of the markers studied with the outcomes of MI, which is also a limitation of the study. In the future, a more detailed study of the dynamics of IL-1β and NLRP3 protein levels at different time periods after MI and a comparison of these data with the development of adverse cardiovascular complications is needed. The data obtained will allow us to potentially identify a group of responders who should receive additional anti-inflammatory therapy at different times after MI. In addition, it will be possible to predict the risk of long-term adverse outcomes from MI based on the early assessment of blood levels of the biomarkers being studied.

It cannot be also excluded that the predominant mechanism of myocardial damage (ischemia–reperfusion) may also influence IL-1β dynamics [18]. Our study included patients with a total duration of myocardial ischemia of up to 24 h and with early revascularization in the IRA basin with the complete restoration of blood flow according to the TIMI (3) scale in 88.8% of cases, which allows us to consider our results in the context of the “reperfused infarction” model.

Unexpectedly, we found an inverse correlation between IL-1β levels and BMI. Previous studies have identified IL-1β as a source of inflammatory response in human adipose tissue, particularly its subcutaneous depot [23], and there is evidence that the IL-1 system is involved in the initiation of obesity in general [24]. However, this excess body weight evaluated by BMI does not reflect the true volume of subcutaneous fat. Waist circumference is a more specific parameter but was not included in our study. Therefore, this conclusion requires confirmation in further studies.

The results of our study have the potential to form the basis of new hypotheses and scientific directions, but they need to be verified by more powerful research. Follow-up studies are required to better understand the pleiotropic effects of pro-inflammatory cytokines in the pathogenesis of different phases of acute MI, which would take into account the timing and mechanisms of ischemic myocardial injury and assess long-term disease outcomes.

### Study Limitations

The weaknesses of our study include the small sample size, the single-center nature of the study, and the lack of evaluation of the long-term prognosis. Only patients with a short duration of myocardial ischemia and the early achievement of reperfusion were included in the study. The instrumental assessment of myocardial ischemic damage was limited to the calculation of echocardiographic parameters, but not to the use of contrast-enhanced cardiac MRI in the early postinfarction period.

## 5. Conclusions

The data from our study describe for the first time the plasma levels of NLRP3 and IL-1β in patients with STEMI in various vascular basins. The concentrations of NLRP3 and IL-1β were higher on the first day of MI with a maximum decrease on the third day. The levels of both markers in venous plasma correlated with those in the peripheral arterial blood and the IRA, allowing their routine determination in venous plasma on the first day after MI. Direct correlations were observed between IL-1β concentrations and WMI, and inverse correlations with LVEF and left ventricular stroke volume, which characterize the contribution to adverse myocardial remodeling after MI. IL-1β levels were inversely correlated with BMI. The assessment of the dynamics of NLRP3 protein and IL-1β levels showed two bidirectional trends in changes in their concentrations during hospitalization: trend 1: initially higher levels with a gradual decrease by day 7; trend 2: initially lower levels followed by a more significant increase by day 7. Trend 1 was associated with the longer duration of myocardial ischemia and higher plasma troponin I levels. The further evaluation of the long-term outcomes of MI will allow identifying inflammatory factors that input to the development of secondary MACE and will provide a new step in the phenotyping of post-MI inflammation and identifying a cohort of patients who will benefit from anti-inflammatory treatment after MI.

## Figures and Tables

**Table 1:** Clinical and anamnestic characteristics of patients.

Parameter	Result
Age, full years	58 [49; 68]
Sex, *n* (%)	31 (69)—men, 14 (31)—women
BMI, kg/m2	27.1 [25.3; 31.5]
GRACE, %	5 [3; 8]
CRUSADE, %	6.1 [5; 8.4]
Time from onset of symptoms to admission, min	125.5 [78.5; 211]
Time door-balloon, min	84 [54; 153]
SBP, mmHg	142 [128; 162]
DBP, mmHg	85 [74; 98]
HR, bpm	81 [67; 92]
Primary/recurrent MI, *n* (%)	39 (86.6)—primary 6 (13.4)—recurrent
Smoking, *n* (%)	28 (62.2)
Hypertension, *n* (%)	45 (100)
Overweight, *n* (%)	19 (42.2)
Obesity, *n* (%)	16 (35.6)
Dyslipidemia, *n* (%)	43 (95.6)
COPD/bronchial asthma, *n* (%)	1 (2.2)
Gastric/duodenal ulcer, *n* (%)	4 (8.9)
CKD, *n* (%)	12 (26.7)
Thrombolysis at the pre-hospital phase, *n* (%)	14 (31.1)
Effective thrombolysis, *n* (%)	4 (8.9)
Infarct-related coronary artery, *n* (%)	23 (51.2)—LAD 15 (33.3)—RCA 4 (8.9)—LCx 1 (2.2)—LMB 1 (2.2)—PDA 1 (2.2)—DA
Atrial fibrillation, *n* (%)	2 (4.4)
Ventricular fibrillation, *n* (%)	4 (8.9)
Premature ventricular complexes, *n* (%)	6 (13.4)
Ventricular tachycardia, *n* (%)	5 (6.7)
Killip I, *n* (%)	32 (71.3)
Killip II, *n* (%)	2 (4.4)
Killip III, *n* (%)	5 (11)
Killip IV, *n* (%)	6 (13.3)
Left ventricular ejection fraction (B-mode), %	53 [43; 58]
Left ventricular mass index, g/m^2^	91.5 [83; 102]
Stroke volume, ml	50 [41; 59]
End-diastolic volume, ml	99 [86; 116]
End-systolic volume, ml	50 [41; 59]

Notes: BMI—body mass index; GRACE—Global Registry of Acute Coronary Events; CRUSADE—Can Rapid risk stratification of Unstable angina patients Suppress ADverse outcomes with Early implementation of the ACC/AHA guidelines; SBP—systolic blood pressure; DBP—diastolic blood pressure; HR—heart rate; MI—myocardial infarction; COPD—chronic obstructive pulmonary disease; CKD—chronic kidney disease; LAD—left-anterior descending coronary artery; RCA—right coronary artery; LCx—left circumflex coronary artery; LMB—left marginal branch; PDA—posterior descending coronary artery; DA—diagonal branch. Quantitative values are presented according to the law of normal distribution of values: median, interquartile range (Me [Q25; Q75]), qualitative values are presented with absolute values (*n*) and relative frequencies (%).

**Table 2:** Laboratory characteristics of patients.

Parameter	1 Day	3 Days	7 Days
Leucocytes, 109/L	10.4 [8.2; 13.7]	8.4 [6.7; 10.2]	7.5 [5.8; 10.4]
Erythrocytes, 1012/L	4.8 [4.3; 5.2]	4.7 [4.3; 5.1]	4.7 [4.4; 5.2]
Hemoglobin, g/L	145 [133; 154]	138.5 [127.5; 153]	143 [124; 157]
Platelets, 109/L	213 [192; 241]	205 [185; 240]	237,5 [201; 312]
CPK, u/L	155 [107; 348]	273 [151; 566]	106 [71; 190]
Troponin I, ng/L	527.9 [47.4; 1480.2]	5492 [2483; 41877.2]	824.3 [252.5; 2966.9]
CPK-MB, u/L	24 [14.7; 67.6]	29.3 [18.9; 39.6]	22.7 [15.7; 32.3]
Glucose, mmol/L	8.9 [7.3; 11.9]	5.8 [5.2; 6.9]	5.7 [5.3; 6.1]
Urea, mmol/L	5.8 [5; 7.6]	6.2 [4.7; 7.1]	5.6 [4.4; 7.2]
Creatinine, μmol/L	88 [77; 101]	91 [84; 102]	88 [77; 103]
GFR, ml/min/1.73 m^2^	84 [69; 97]	81 [67; 89]	-
AST, u/L	40 [24; 69]	66 [38; 94]	34 [22; 52]
ALT, u/L	24 [19; 46]	38 [28; 57]	39 [23; 60.5]
HDL-C, mmol/L	1.1 [1; 1.3]	-	-
LDL-C, mmol/L	3.4 [2.7; 4]	-	-
Cholesterol, mmol/L	5.2 [4.4; 5.8]	-	-
Triglycerides, mmol/L	1.2 [0.8; 1.9]	-	-
CRP, mg/L	10.7 [6.7; 31.2]	29.3 [10.7; 75.3]	16.1 [6.5; 28.5]

Note: CPK—creatine phosphokinase; GFR—glomerular filtration rate; AST—aspartate aminotransferase; ALT—alanine aminotransferase; HDL—high-density lipoprotein cholesterol; LDL-C—low-density lipoprotein cholesterol; CRP—C-reactive protein. Values are presented according to the law of the normal distribution of values: median, interquartile range (Me [Q25; Q75]).

**Table 3:** NLRP3 protein and IL-1β plasma levels of patients.

	Day 1			Day 3	Day 7	
	0 IRA	1 PA	2 V	3 V	4 V	*p*-Value
NLRP3, ng/mL	3.6 [2.1; 7.2]	3.8 [2; 7]	4.3 [2.8; 5.3]	2.5 [1.6; 3.7]	2.9 [2.1; 4]	p0-3 = 0.033 p2-3 = 0.014 p2-4 = 0.025
IL-1β, pg/mL	12.7 [7; 17.3]	12 [8.6; 17.5]	11.9 [8.1; 19.9]	7.9 [5.3; 17.3]	11 [5,3; 21,4]	p1-3 = 0.001 p2-3 = 0.002

Note: NLRP3—Nod-like receptor family pyrin domain containing 3; IL-1β—interleukin 1 beta; IRA—infarct-related coronary artery; PA—peripheral artery; V—peripheral vein. Values are presented according to the law of normal distribution of values: median, interquartile range (Me [Q25; Q75]). Intergroup comparisons are indicated for statistically significant *p* values (<0.05).

**Table 4:** Comparison of basic characteristics of patients according to the dynamics of inflammatory markers in venous blood plasma at 7 days.

	Group 1, IL-1β ↓ (*n* = 21)	Group 2, IL-1β ↑ (*n* = 15)	*p*-Value	Group 3, NLRP3 ↓ (*n* = 30)	Group 4, NLRP3 ↑ (*n* = 12)	*p*-Value
Age, years	60.5 [52; 67.5]	55 [45; 71]	0.647	57 [48; 68]	64 [53; 67]	0.425
Male sex, *n* (%)	22 (81.4)	10 (55.5)	0.091	22 (68.8)	10 (76.9)	0.595
Primary MI, *n* (%)	23 (85.1)	17 (94.4)	0.496	30 (93.4)	10 (76.9)	0.468
GRACE, %	5.5 [3; 8]	5 [2; 13]	0.616	5 [3; 8]	6 [5; 8]	0.647
Total time of myocardial ischemia, min	281 [157; 556]	168.5 [111; 331]	0.048	250 [142; 394]	213 [162; 492]	0.885

Note: MI—myocardial infarction; GRACE—Global Registry of Acute Coronary Events. Values are presented according to the law of normal distribution of values: median, interquartile range (Me [Q25; Q75]). Statistically significant results (*p* < 0.05) are highlighted in red font. Symbols ↓ or ↑ represent the main trends in marker concentrations on the 7th day of hospitalization.

**Table 5:** Comparison of laboratory and instrumental parameters of myocardial damage as a function of the dynamics of inflammatory markers in venous blood plasma at 7 days.

	Group 1, IL-1β ↓ (*n* = 21)	Group 2, IL-1β ↑ (*n* = 15)	*p*-Value	Group 3, NLRP3 ↓ (*n* = 30)	Group 4, NLRP3 ↑ (*n* = 12)	*p*-Value
Laboratory parameters						
Troponin I, ng/L, day 1	536 [68.4; 3566.8]	511 [42.6; 1480]	0.510	527 [62.9; 1480]	545 [46.5; 1072.5]	0.579
CPK-MB, u/L, day 1	23 [15.7; 65.8]	38.7 [13.5; 68]	0.495	25 [15.7; 68]	23.2 [13.6; 61.3]	0.877
Troponin I, ng/L, day 3	15859 [2262.8; 25000]	4198 [2511; 9262]	0.047	7024 [3040; 25000]	3181 [1641; 10555]	0.148
CPK-MB, u/L, day 3	38 [19.1; 45]	24.6 [18.6; 29.9]	0.100	29 [18.9; 41.6]	29.3 [18; 42.9]	0.611
Troponin I, ng/L, day 7	2966 [603.8; 4391.9]	448 [143.5; 1179]	0.003	1147 [395; 3110]	665 [222; 2255]	0.46
CPK-MB, u/L, day 7	23 [16.8; 31.7]	22.3 [15.1; 32.9]	0.748	21.6 [16.3; 29.1]	23.8 [13.2; 110]	0.8
Instrumental parameters						
EDV, ml	96 [86; 126]	99 [80; 110]	0.558	99 [82; 117]	98 [94.5; 112.5]	0.534
ESV, ml	54.5 [38; 71]	45 [37; 53]	**0.083**	50.5 [37.5; 64]	45 [37; 54.5]	0.686
LVEF (B-mode), %	48 [36; 55]	54 [46; 61]	**0.075**	49.5 [41.5; 57]	54 [49; 59.5]	0.354
SV, ml	48 [38; 56]	52 [43; 60]	0.400	47.5 [39.5; 58.5]	53 [47.5; 59.5]	0.185
CI, l/min/m	1.9 [1.4; 2.3]	2.1 [1.6; 2.4]	0.249	1.9 [1.5; 2.4]	2 [1.65; 2.25]	0.989
WMI	1.57 [1.2; 2]	1.31 [1; 1.6]	**0.083**	1.44 [1.12; 1.88]	1.38 [1.12; 1.69]	0.83

Note: CPK—creatine phosphokinase; EDV—end-diastolic volume; ESV—end-systolic volume; LVEF—left ventricular ejection fraction; SV—stroke volume; CI—cardiac index; WMI—wall motion index. Values are presented according to the law of normal distribution of values: median, interquartile range (Me [Q25; Q75]). Statistically significant results (*p* < 0.05) are highlighted in red font. Statistical tendency (0.05 ≤ *p* < 0.10) are highlighted in bold font. Symbols ↓ or ↑ represent the main trends in marker concentrations on the 7th day of hospitalization.

**Table 6:** Relationship between NLRP3 protein and IL-1β concentrations of arterial and venous blood plasma at different times of myocardial infarction.

	IL-1β pg/mL, IRA	IL-1β pg/mL, PA	IL-1β pg/mL, V1	IL-1β pg/mL, V3	IL-1β pg/mL, V7	NLRP3 ng/mL, IRA	NLRP3 ng/mL, PA	NLRP3 ng/mL, V1	NLRP3 ng/mL, V3	NLRP3 ng/mL, V7
IL-1β pg/mL, IRA	1.0000									
IL-1β pg/mL, PA	0.7745	1.0000								
IL-1β pg/mL, V1	0.6587	0.5806	1.0000							
IL-1β pg/mL, V3	0.7225	0.7330	0.6865	1.0000						
IL-1β pg/mL, V7	0.5334	0.6083	0.5962	0.7884	1.0000					
NLRP3 ng/mL, IRA	0.3050	0.3511	0.2381	0.3135	0.2148	1.0000				
NLRP3 ng/mL, PA	0.3299	0.4038	0.3359	0.3650	0.2036	0.7933	1.0000			
NLRP3 ng/mL, V1	0.2993	0.2866	0.2383	0.1390	0.0348	0.5666	0.6826	1.0000		
NLRP3 ng/mL, V3	−0.0138	0.0493	0.1030	0.0370	0.1622	0.1606	0.2552	0.3587	1.0000	
NLRP3 ng/mL, V7	0.2656	0.1745	0.0720	0.1544	0.0741	0.3317	0.3747	0.4500	0.4079	1.0000

Note: NLRP3—Nod-like receptor family pyrin domain containing 3; IL-1β—interleukin 1 beta; IRA—infarct-related coronary artery; PA—peripheral artery; V1—peripheral vein, day 1; V3—peripheral vein, day 3; V7—peripheral vein, day 7. Numerical values of correlations (r) are indicated, with statistically significant results (*p* < 0.05) highlighted in red.

## Data Availability

The datasets are stored in closed access in the form of a personalized electronic summary table created using Excel 2010. The datasets used and/or analyzed in the current study are available from A. Nesova upon reasonable request.

## Abbreviations

BMI—body mass index; CHD—coronary heart disease; CKD—chronic kidney disease; CRP—C-reactive protein; CS—cardiogenic shock; CVD—cardiovascular diseases; ESV—end-systolic volume; GRACE—Global Registry of Acute Coronary Events; IL-1β—interleukin 1 beta; IL-6—interleukin 6; IL-18—interleukin 18; IRA—infarct-related coronary artery; LVEF—left ventricular ejection fraction; MACE—major adverse cardiac events; MI—myocardial infarction; MRI—magnetic resonance imaging; NLRP3—Nod-like receptor family pyrin domain containing 3; PCI—percutaneous coronary intervention; STEMI—ST elevation myocardial infarction; SV—stroke volume; TIMI—Thrombolysis In Myocardial Infarction; WMI—wall motion index.
